# Supplementary material for: The Minus-End-Directed Kinesin OsDLK Shuttles to the Nucleus and Modulates the Expression of Cold-Box Factor 4
Source: Int J Mol Sci. 2022 Jun 3;23(11):6291. doi: 10.3390/ijms23116291 (PMC9181729; doi:10.3390/ijms23116291)
Supplement: Supplementary file 1 [file ijms-23-06291-s001.zip › ijms-1687931-supplementary.pdf]

The Minus-End-Directed Kinesin OsDLK Shuttles to the Nucleus and Modulates the Expression of Cold-Box Factor 4

Xiaolu Xu[1,*], Sabine Hummel[2], Klaus Harter[2], Üner Kolukisaoglu[2], Michael Riemann[1], Peter Nick[1, *]

Contents

Suppl. Fig. S1 2

Suppl. Fig. S2 3

Suppl. Fig. S3 4

Suppl. Fig. S4 6

Suppl. Table S1.......... 7

Suppl. Table S2.......... 8

Suppl. Table S3.......... 9

Suppl. Fig. S1

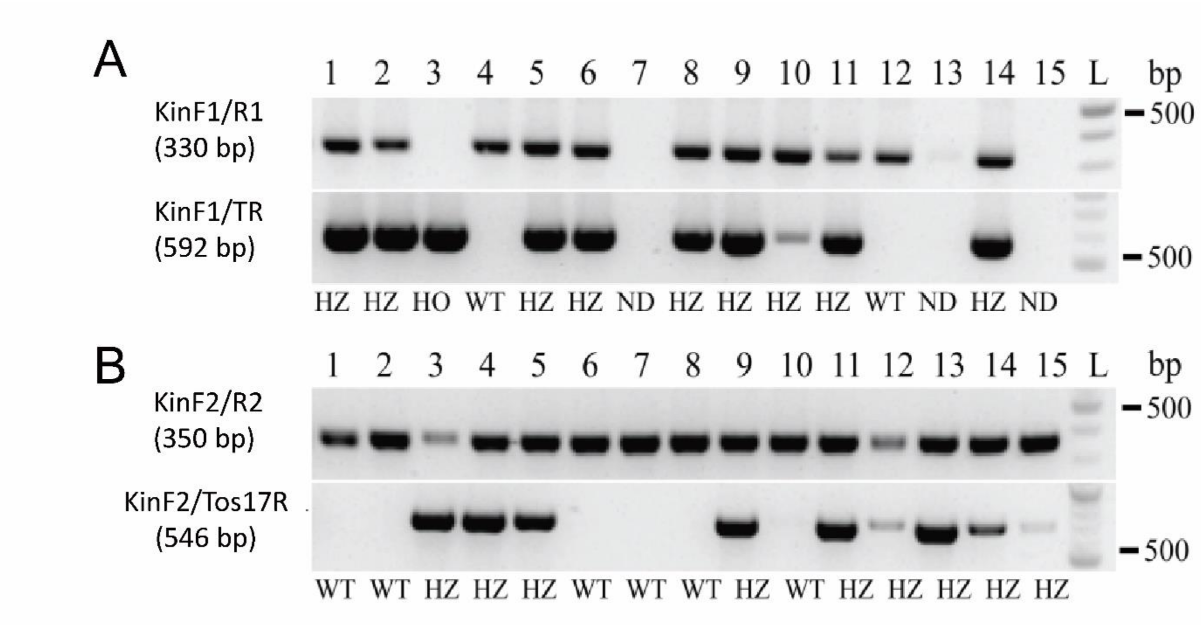

Figure S1. Representative gel electrophoresis results from PCR amplification of genotyping rice insertion lines. **A** Representative PCR genotyping results from 15 samples of genomic DNA extracted from T-DNA insertion line PFG_3A-07110.R as templates. Two rounds of PCR were carried out with two pairs of primer which were genome-specific (KinF1/R1, upper row) or insertion-specific (KinF1/TR, lower row), respectively. **B** Representative genotyping results for 16 samples of genomic DNA from Tos−17 insertion line ND4501_0_508_1 using genome specific (KinF2/R2, upper row) and insertion-specific (KinF2/Tos17R, lower row) primer combinations, respectively.

Suppl. Fig. S2

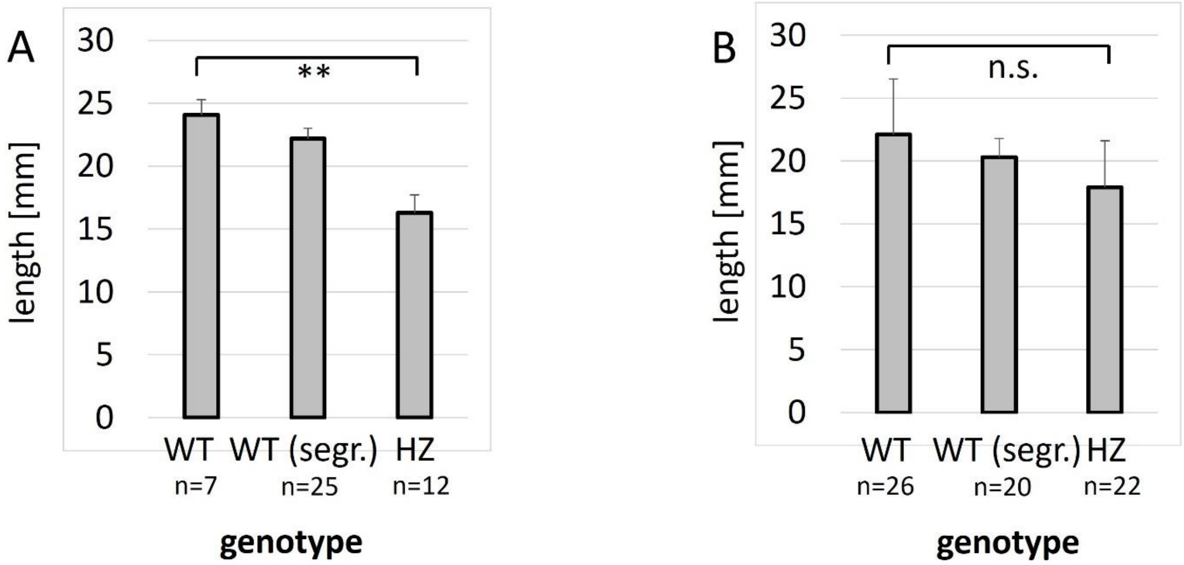

Figure S2. Relationship between DLK and elongation of the fully expanded second leaf sheath. Mean length of fully expanded etiolated coleoptiles in the Tos−17 insertion line ND4501_0_508_1A (**A**) and the T-DNA insertion line PFG_3A-07110.R (**B**). WT gives the values for the respective background (‚Nipponbare' in **A**, ‚Dongjin' in **B**), WT segr. for segregating plants genotyped as WT, HZ for the genotyped heterozygotes. No homozygous mutants were recovered at this stage. Error bars represent SE, ns not significant at *P*<5%, ** significant at *P*<1% based on a homoskedastic t-test. The number of individuals per set is indicated below the respective bar.

Suppl. Fig. S3

```
AthCBF4 B2BJ26   MNPFYSTFPDS---FLSISDHRSP---VSDSSECSPKLASSCPKKRAGRKKFRETRHPIY 54
AthCBF1 B6DTR3   MNSF-SAFSEM-----FGSDYEP------QGGDYCPTLATSCPKKPAGRKKFRETRHPIY 48
AthCBF2 B2BIW9   MNSF-SAFSEM-----FGSDYESP---VSSGGDYSPKLATSCPKKPAGRKKFRETRHPIY 51
AthCBF3 B2BIZ3   MNSF-SAFSEM-----FGSDYESS---VSSGGDYIPTLASSCPKKPAGRKKFRETRHPIY 51
OsDREB1F Q8S9Z5  MDT----------EDTSSASSSSVSPPSSPGGG---HHHRLPPKRRAGRKKFRETRHPVY 47
NtAvr9 Q9FQZ8    MDIFRSYYSDPLAEYSSISDSSSSSCNRANHSDEEVMLASNNPKKRAGRKKFRETRHPVY 60
                 *:                :.           .          **: ************:*

AthCBF4 B2BJ26   RGVRQRNS-GKWVCEVREPNKKSRIWLGTFPTVEMAARAHDVAALALRGR-SACLNFADS 112
AthCBF1 B6DTR3   RGVRQRNS-GKWVSEVREPNKKTRIWLGTFQTAEMAARAHDVAALALRGR-SACLNFADS 106
AthCBF2 B2BIW9   RGVRQRNS-GKWVCELREPNKKTRIWLGTFQTAEMAARAHDVAAIALRGR-SACLNFADS 109
AthCBF3 B2BIZ3   RGVRRRNS-GKWVCEVREPNKKTRIWLGTFQTAEMAARAHDVAALALRGR-SACLNFADS 109
OsDREB1F Q8S9Z5  RGVRARAGGSRWVCEVREPQAQARIWLGTYPTPEMAARAHDVAAIALRGERGAELNFPDS 107
NtAvr9 Q9FQZ8    RGVRKRNS-DKWVCELREPNKKSRIWLGTFPSAEMAARAHDVAAIALRGR-SACLNFADS 118
                 **** * . .:**.*:***: ::******: : ***********:****. .* *** **

AthCBF4 B2BJ26   AWRLRIPETTCPKEIQKAASEAAMAFQNETTTEGSKTA-AEAEEAAGEGVREGERRAEEQ 171
AthCBF1 B6DTR3   AWRLRIPESTCAKDIQKAAAEAALAFQDETCDTTTTNHGLDMEETM-----VEAIYTPEQ 161
AthCBF2 B2BIW9   AWRLRIPESTCAKEIQKAAAEAALNFQDEMCHMTTDAHGLDMEETL-----VEAIYTPEQ 164
AthCBF3 B2BIZ3   AWRLRIPESTCAKDIQKAAAEAALAFQDEMCDATT-DHGFDMEETL-----VEAIYTAEQ 163
OsDREB1F Q8S9Z5  PSTLPRARTASPEDIRLAAAQAAELYRRPPPPLA-------LPEDPQEGTSGGGATATSG 160
NtAvr9 Q9FQZ8    AWKLPIPASTDAKDIQKAAAEAAEAFRSSEAENMPEYSGEDTK------------EVNST 166
                    *    ::  ::*: **::**  ::                             . .

AthCBF4 B2BJ26   NGGVFYMDDEALLGMPNFFENMAEGMLLPPPEVGWNHNDF--DGV------GDVSLWSFD 223
AthCBF1 B6DTR3   SEGAFYMDEETMFGMPTLLDNMAEGMLLPPPSVQWNHNYDGEGD-------GDVSLWSY- 213
AthCBF2 B2BIW9   SQDAFYMDEEAMLGMSSLLDNMAEGMLLPSPSVQWNYNFDVEGD-------DDVSLWSY- 216
AthCBF3 B2BIZ3   SENAFYMHDEAMFEMPSLLANMAEGMLLPLPSVQWNHNHEVDGDD------DDVSLWSY- 216
OsDREB1F Q8S9Z5  RPAAVFVDEDAIFDMPGLIDDMARGMMLTPPAIGRSLDDWAAIDDDDDHYHMDYKLWMD- 219
NtAvr9 Q9FQZ8    PENMFYMDEEALFFMPGLLVNMAEGLMLPPPQCSQIGDHM--EAD------VDMPLWSYS 218
                     .::.::::: *  :: :**.*::*  *      :              *  **

AthCBF4 B2BJ26   E 224
AthCBF1 B6DTR3   -
AthCBF2 B2BIW9   -
AthCBF3 B2BIZ3   -
OsDREB1F Q8S9Z5  -
NtAvr9 Q9FQZ8    I 219
```

Apetala2/Ethylene Response Factor domain https://prosite.expasy.org/doc/PS51032

CBF signatures according to Haake 2002

Polar bias

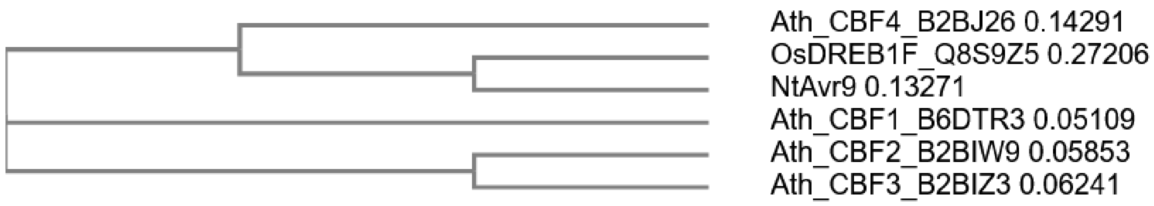

Figure S3. Sequence analysis of NtCf9 and other C-repeat-binding factors (CBFs).

Suppl. Fig. S4

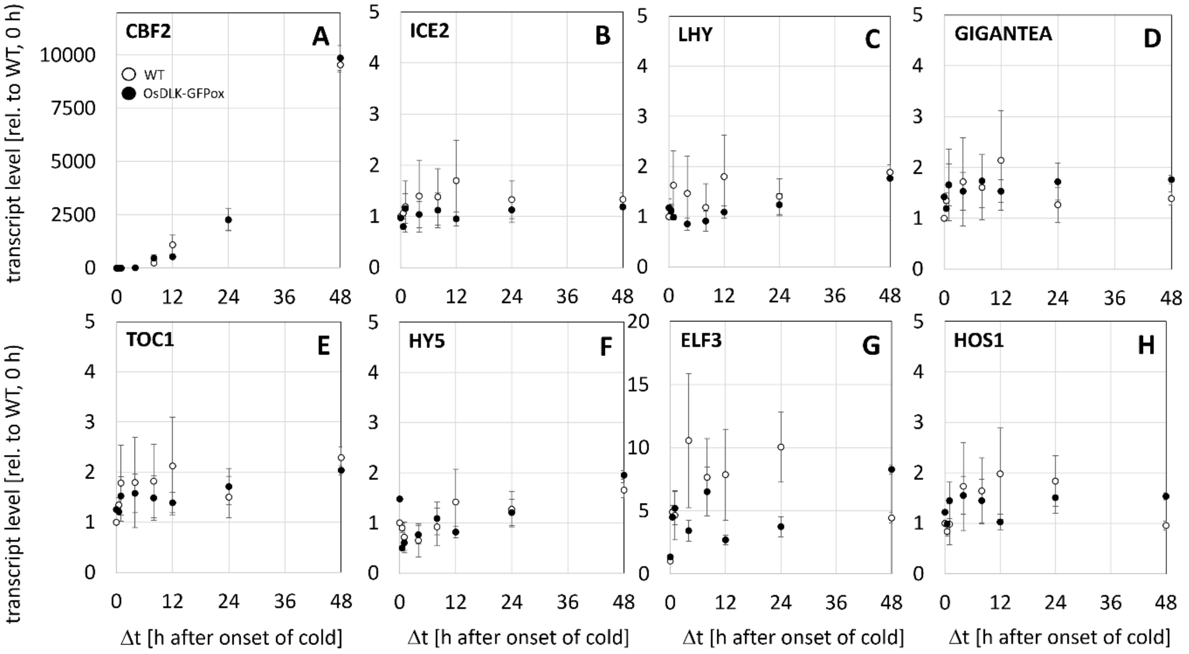

Figure S4. Time course of steady-state transcript levels under continuous cold stress (0°C) for different genes involved in the regulation of cold responses in non-transformed tobacco BY−2 cells (WT, open circles) and in cells expressing OsDLK-GFP under control of the CaMV 35S promoter (OsDLK-GFPox, closed circles). Data represent means and standard error from two independent experimental series with three technical replications per set. The transcripts were A Cold Box Factor 2 (CBF2), a transcriptional activator of cold-responsive genes, B Inducer of CBF expression (ICE2), the master switch for CBFs, C Late Elongated Hypocotyl, a regulator of CBFs acting in parallel of ICE, D Gigantea, a positive regulator of freezing tolerance acting independently of CBFs, E Timing of Cab Expression 1 (TOC1), a phytochrome dependent repressor of CBF expression, F Hypocotyl 5 (HY5), a light-dependent regulator of cold acclimation acting independentyl of CBFs, G Early Flowering 3 (ELF3) a phytochrome dependent regulator of CBFs H High Expression of Osmotically Responsive Genes (HOS1) a negative regulator of CBFs. Data are normalised to the resting level in the wild type.

Suppl. Table S1

Table S1. Oligonucleotid primers used for genotyping of rice mutants bearing T-DNA and Tos−17 insertion in the OsDLK locus.

name	sequence	target
TR	5′-CATCGAAACGCAGCACGATACGC-3′	insert-specific for T-DNA insertion line PFG_3A-07110.R
Tos17R	5′-CTGTATAGTTGGCCCATGTCCAG-3′	insert-specific for Tos−17 insertion line, ND4501_0_508_1A
KinF1	5′-ATCTGCTAGCCACTAATCGCAC-3′	upstream for T-DNA insertion line PFG_3A-07110.R
KinR1	5′-TTATGCTTCTAACTTCACTTCAGGC-3′	downstream for T-DNA insertion line PFG_3A-07110.R
KinF2	5′-GATTTTGTTCCCCTTGGACA-3′	upstream for Tos−17 insertion line ND4501_0_508_1A
KinR2	5′-GCTCGTACAATCAACAAAGCC-3′	downstream for Tos−17 insertion line ND4501_0_508_1A

Suppl. Table S2

Table S2. Oligonucleotid primers used to measure expression of OsDLK in rice plants. Annealing temperature was 60°C throughout.

designation	sequence
Ubiquitin 10 fw	5′-GAGCCTCTGTTCGTCAAGTA-3′
Ubiquitin 10 rv	5′-ACTCGATGGTCCATTAAACC-3′
GAPDH fw	5′-CTGATGATATGGACCTGAGTCTACTTTT-3′
GAPDH rv	5′-CAACTGCACTGGACGGCTTA-3′
qDLK fw	5′-AGATTTCCCAACTCATCCAA-3′
qDLK rv	5′-ATCCTTTCTGGTCATGCAAT-3′

Suppl. Table S3

Table S3. Oligonucleotid primers for cold-stress related genes expression in tobacco BY–2 cells. Annealing temperature was 58°C throughout [16].

name	sequence	target
L25 fw	5′-GTTGCCAAGGCTGTCAAGTCAGG-3′	housekeeping gene L25
L25 rv	5′-GCACTAATACGAGGGTACTTGGGG-3′	housekeeping gene L25
EF1α fw	5′-TGAGATGCACCACGAAGCTCTTC-3′	housekeeping gene EF1α
EF1α rv	5′-GCTGAAGCACCCATTGCTGGG-3′	housekeeping gene EF1α
CBF2 fw	5′-CTCTACTAGCATCAGAAAGTGT-3′	tobacco CBF2
CBF2 rv	5′-ACTTGCCTAACCAAGTCAT-3′	tobacco CBF2
Cf9 fw	5′-AAGAGGAATTCAGACAAGTG-3′	tobacco CBF4
Cf9 rv	5′-AAAGTTCAAGCAAGCAGAAC-3′	tobacco CBF4
TOC1 fw	5′-AAGAAATCCTCTGCTCTCAC-3′	tobacco TOC1
TOC1 rv	5′-CGATTAACTTCTCCGGTCCA-3′	tobacco TOC1
HY5 fw	5′-TGTAGGTAAGGCCGAGAT-3′	tobacco HY5
HY5 rv	5′-ATCACTCTCCATACCTTCACA-3′	tobacco HY5
Gl fw	5′-ACAGCTAGAGCAGTACAAC-3′	tobacco Gl
Gl rv	5′-CGAACTGTGGCTGGTAAG-3′	tobacco Gl
LHY fw	5′-TGGAGATGCTGGGAATCG-3′	tobacco LHY
LHY rv	5′-GGCAACTTCTCTCTGGTG-3′	tobacco LHY
HOS1 fw	5′-TGAGATTAGCGATTTGAGGC-3′	tobacco HOS1
HOS1 rv	5′-ATCAAGGTCAGTTTACGCA-3′	tobacco HOS1
ELF3 fw	5′-TCCTTCTCAACCACACAGTTTA-3′	tobacco ELF3
ELF3 rv	5′-GTAGTTCAAACACTTGTATCGC-3′	tobacco ELF3
